# Genome-wide association study of plant color in *Sorghum bicolor*

**DOI:** 10.3389/fpls.2024.1320844

**Published:** 2024-04-10

**Authors:** Lihua Wang, Wenmiao Tu, Peng Jin, Yanlong Liu, Junli Du, Jiacheng Zheng, Yi-Hong Wang, Jieqin Li

**Affiliations:** ^1^ College of Agriculture, Anhui Science and Technology University, Fengyang, Anhui, China; ^2^ Anhui Province International Joint Research Center of Forage Bio-breeding, Chuzhou, China; ^3^ Department of Biology, University of Louisiana at Lafayette, Lafayette, LA, United States

**Keywords:** GWAS, plant color, resequencing, sorghum, SNP

## Abstract

**Introduction:**

Sorghum plant color is the leaf sheath/leaf color and is associated with seed color, tannin and phenol content, head blight disease incidence, and phytoalexin production.

**Results:**

In this study, we evaluated plant color of the sorghum mini core collection by scoring leaf sheath/leaf color at maturity as tan, red, or purple across three testing environments and performed genome-wide association mapping (GWAS) with 6,094,317 SNPs markers.

**Results and Discussion:**

Eight loci, one each on chromosomes 1, 2, 4, and 6 and two on chromosomes 5 and 9, were mapped. All loci contained one to three candidate genes. In *qPC5-1*, Sobic.005G165632 and Sobic.005G165700 were located in the same linkage disequilibrium (LD) block. In *qPC6*, Sobic.006G149650 and Sobic.006G149700 were located in the different LD block. The single peak in *qPC6* covered one gene, Sobic.006G149700, which was a senescence regulator. We found a loose correlation between the degree of linkage and tissue/organ expression of the underlying genes possibly related to the plant color phenotype. Allele analysis indicated that none of the linked SNPs can differentiate between red and purple accessions whereas all linked SNPs can differentiate tan from red/purple accessions. The candidate genes and SNP markers may facilitate the elucidation of plant color development as well as molecular plant breeding.

## 1 Introduction

Plant color in sorghum [*Sorghum bicolor* (L.) Moench] is defined as the stem/leaf sheath/leaf color (Rana et al., 1976; Reddy et al., 2008; Rooney, 2016; Fedenia et al., 2020) at maturity (Valencia and Rooney, 2009). Plant color is controlled by the *P* and *Q* genes. A sorghum plant with *P_Q*_ genotype is purple, whereas *P_ qq* is red and *pp Q*_ and *pp qq* are tan (Dykes et al., 2009; Valencia and Rooney, 2009; Dykes et al., 2011).

Plant color is associated with other phenotypes or consumer preferences. For example, white sorghum grain from tan plants is more desirable for human or animal consumption (Williams-Alanis et al., 1999; Funnell and Pedersen, 2006; Rooney, 2016). This is probably because tan plants tend to have lower tannin content compared with purple plants (Gourley and Lusk, 1978; Dykes et al., 2005). However, sorghum grains grown on plants with purple/red plant color do have higher levels of total phenols than those from tan plants (Dykes et al., 2005), although grains from some tan plants have the highest flavone (luteolin and apigenin) content (Dykes et al., 2009, Dykes et al., 2011). Tan plants tend to have lower head blight incidence caused by *Fusarium moniliforme* than red plants (Torres-Montalvo et al., 1992), but it is not clear if this is related to the high luteolin and apigenin contents. Du et al. (2010) have shown that flavones such as luteolin function as a phytoalexin against the sorghum anthracnose pathogen *Colletotrichum sublineolum*.

Sorghums with red/purple plant color produce the highest levels of 3-deoxyanthocyanidins (apigeninidin and luteolinidin) (Dykes et al., 2011), which are also phytoalexins induced by fungal attack (Snyder and Nicholson, 1990). The purple phenotype after fungal attack is determined by the production of two 3-deoxyanthocyanidins, apigeninidin and luteolinidin, which are not produced by the tan plants (Kawahigashi et al., 2016). The underlying *P* gene has been cloned using map-based cloning in progeny from a cross between purple Nakei-MS3B (*PP*) and tan Greenleaf (*pp*) cultivars; the gene was located in a 27-kb genomic region between markers CA29530 and SB25792 on chromosome 6. Four candidate genes identified in this region were similar to the maize leucoanthocyanidin reductase gene induced by wounding, and only the Sb06g029550 gene was induced in both cultivars after wounding. The Sb06g029550 protein was detected in Nakei-MS3B but only slightly in Greenleaf. A recombinant Sb06g029550 protein had a specific flavanone 4-reductase activity and converted flavanones (naringenin or eriodictyol) to flavan-4-ols (apiforol or luteoforol) *in vitro* (Kawahigashi et al., 2016).

In this study, we evaluated plant color of the sorghum mini core collection (MC; Upadhyaya et al., 2009) as the association panel. This panel has been extensively characterized, such as its genetic structure and linkage disequilibrium (Wang et al., 2013) and effectiveness for association mapping (Upadhyaya et al., 2013). Most importantly, the panel has been used to clone a pleiotropic *SbSNF4-2* (*SnRK1βγ2*) that increases both biomass and sugar yield in sorghum and sugarcane (Upadhyaya et al., 2022). We scored leaf sheath/leaf color at maturity as tan, red, or purple across three testing environments in Tengqiao/Hainan and Fengyang/Anhui, China, performed association mapping with 6,094,317 SNP markers (Wang et al., 2021), and identified candidate genes strongly linked to plant color.

## 2 Materials and methods

### 2.1 Plant materials and phenotyping

The accessions of the sorghum MC (Upadhyaya et al., 2009, **Table S1**) were grown in Tengqiao, Hainan, China, for two seasons (2021 and 2022) and in Fengyang, Anhui, China, for one season (2022). In both 2021 and 2022 in Tengqiao, Hainan, the plants were grown with a row spacing of 65 cm and a plant spacing within each row of 25 cm. A compound fertilizer (N:P:K = 15:15:15) and urea were applied before planting at 200 kg/ha and 120 kg/ha, respectively. The plot was irrigated once at seedling and once at stem elongation stages and weeded at before three-leaf, during four-to-six-leaf, and before anthesis stages. Pesticides were applied three times to control cutworms, aphids, and honeydew moths.

In Fengyang, Anhui in 2022, the plants were grown with a row spacing of 50 cm and plant spacing within each row of 25 cm. A compound fertilizer (N:P:K = 15:15:15) and urea were applied before planting at 180 kg/ha and 90 kg/ha, respectively. The plot was irrigated once at seedling and once at stem elongation stages and weeded at before three-leaf, during four-to-six-leaf, and before anthesis stages. Pesticides were applied three times to control cutworms, aphids, and honeydew moths.

**Figure 1:**
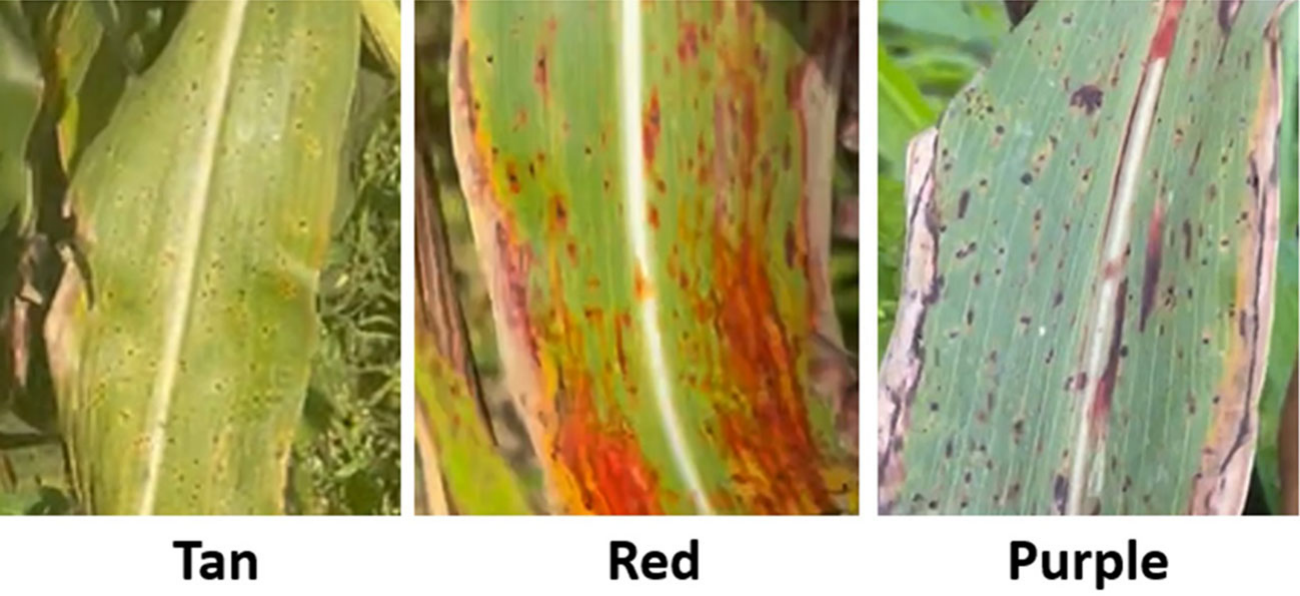
The sorghum plant color phenotype from the mini core collection: tan, red, and purple according to Rooney (2016).

At maturity in all three environments, plant color was scored for leaf/leaf sheath color as “1” (tan), “2” (red), or “3” (purple) (**Figure 1**) according to Rooney (2016).

### 2.2 Genome-wide association study

Genome resequencing of 237 MC accessions (**Supplementary Table S1**) and genome-wide association study (GWAS) were as described in Wang et al. (2021). GWAS was performed with 6,094,317 SNPs from Wang et al. (2021). The kinship matrix (*K*) was generated by EMMAX (Kang et al., 2010), which was used to perform GWAS analyses with the Q matrix calculated using STRUCTURE 2.3.4 (Pritchard et al., 2000) as the covariate variable. The modified Bonferroni correction was used to determine the genome-wide significance thresholds of the GWAS, based on a nominal level of α = 0.05 which corresponds to a *P* value of 8.2E-09, or −log_10_(*P*) values of 8.08. At α = 0.01, these were 1.6E-09 and 8.78, respectively.

### 2.3 Candidate gene identification and allelic effect of linked SNPs

Candidate genes were identified using the reference genome Sorghum bicolor v3.1.1 (Paterson et al., 2009; McCormick et al., 2018) curated at Phytozome (Goodstein et al., 2012) 13 (https://phytozome-next.jgi.doe.gov/). RNA-seq data (McCormick et al., 2018) for each candidate genes were downloaded from the site and provided as **Supplementary Table S2**. To determine the allelic effect of selected SNPs linked to plant color, SNPs in each locus or two loci were grouped together. Only accessions with less than 5% missing data rate for each group of SNPs were included. The original data are provided in **Supplementary Tables S3**-**S8**.

## 3 Results

### 3.1 Phenotype analysis

As described in the Introduction, plant color is controlled by multiple genes. This is reflected in phenotyping in this study. All accessions were consistently scored as either tan (9 accessions) or pigmented (228 accessions) in all three environments (2021_HN, 2022_HN, 2022_FY; **Supplementary Table S1**). However, 47 of the 228 accessions (20.6%) could not be consistently scored as either red or purple across the three environments. This indicates that the trait may be affected by the environment as well as the combinations of multiple genes.

### 3.2 Association mapping

**Figure 2:**
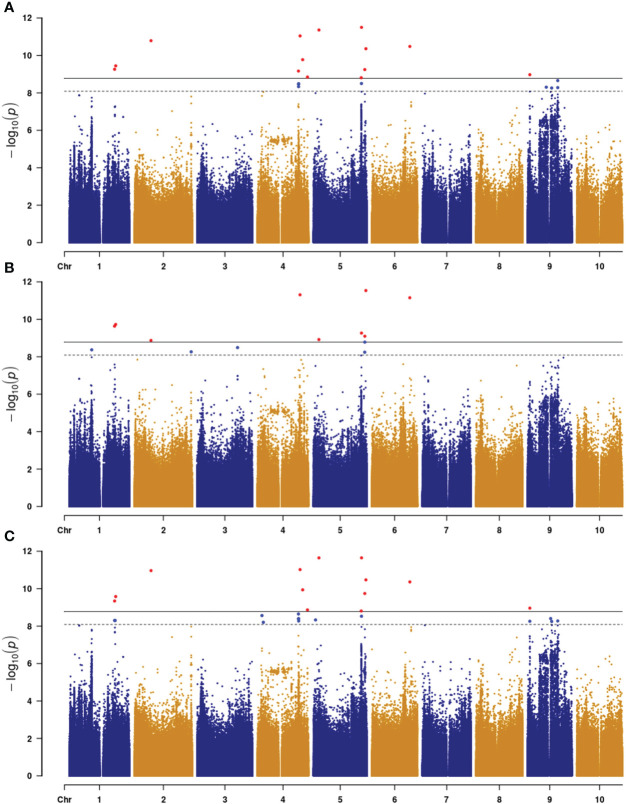
Manhattan plot of plant color with three environments in sorghum (**(A)**, 2021_HN; **(B)**, 2022_HN; **(C)**, 2022_FY**).** Horizontal dash and gray lines indicate the threshold −log(*P*) value at α = 0.05 and 0.01, respectively.

To identify SNP markers linked to the trait, we used the following criteria: 1) more than one marker associated with plant color and at least one of the markers had −log_10_(*P*) higher than the threshold (Upadhyaya et al., 2022), and 2) association had to be present across all three environments (2021_HN, 2022_HN, and 2022_FY). Based on these criteria, we identified eight loci distributed on chromosomes 1, 2, 4, 5, 6, and 9 (**Figure 2**; **Supplementary Figure S1**; **Table 1**). These loci contained 2 (*qPC2* and *qPC4*) to 21 SNP markers (*qPC5-2*) (**Table 1**). The strongest association was with the SNP (64621753) marker on chromosome 5 (*qPC5-2*), with −log_10_(*P*) values of 11.50 in 2021_HN, 11.65 in 2022_HN, and 9.26 in 2022_FY (**Table 1**), respectively. This was followed by the locus on chromosome 6 for 51113980 (*PC6*) with −log_10_(*P*) values of 10.4, 11.1, and 10.3, respectively (**Table 1**). *qPC5-1* and *qPC5-2* were mapped with the most SNPs with −log(*P*) values higher than 6.0, 20, and 21 SNPs (**Table 1**), respectively.

**Table 1:** The plant color QTLs in sorghum detected in all three environments*.

SNPs	−log(*P*)			Candidate gene position	Candidate gene expression**
	HN 2021	HN 2022	FY 2022		
Chr01 (*qPC1*)					
61215127	6.84	7.92	6.51	Sobic.001G324900 DUF2215 Chr01:61207155.61212055 forward 3 kb from 61215127 Sobic.001G325000 disease resistance protein Chr01:61220311.61222354 forward 5 kb from 61215155	Highest expression in leaves Highly expressed in seeds and roots
61215130	7.25	**8.30**	6.28		
61215132	7.25	**8.30**	6.28		
61215133	7.25	**8.30**	6.28		
61215136	7.25	**8.30**	6.28		
61215145	6.82	7.69	6.79		
61215155	6.75	7.66	7.42		
Chr02 (*qPC2*)					
76356664	7.80	7.97	3.89	Sobic.002G416400 bHLH033 Chr02:76362451.76364029 forward Between the SNPs	Highest exp in internode and leaf sheath
76366175	7.44	7.43	**8.26**		
Chr04 (*qPC4*)					
55131019	**9.17**	**8.65**	7.29	Sobic.004G200700 ABI4 Chr04:55121061.55121994 forward 9 kb from 55131019	Highly expressed in panicles
55132948	7.27	7.49	3.01		
Chr05 (*qPC5-1*)					
64207536	7.19	6.90	6.16	Sobic.005G165632 unknown Chr05:64212726.64213572 reverse 64213028 in 5′-UTR and 64213268 in the intron Sobic.005G165700 Plant antimicrobial peptide (MBP-1 family protein precursor) Chr05:64214229.64216294 forward 64216240 in 3′-UTR Sobic.005G165800 MSS1/GTP-binding protein Chr05:64217561.64225842 reverse 64224755 in second intron	Highly expressed in panicles and seeds Panicle and seed specific expression Highly expressed in panicles and leaves
64209111	7.10	6.79	6.09		
64209805	7.10	6.79	6.09		
64209962	7.06	6.76	6.07		
64210891	7.04	6.74	6.04		
64210945	7.33	7.05	6.09		
64211162	7.10	6.79	6.09		
64211239	7.10	6.79	6.09		
64211411	7.10	6.79	6.09		
64211531	7.10	6.79	6.09		
64213028	6.98	6.66	6.17		
64213268	6.55	6.22	4.21		
64216240	7.02	6.97	6.16		
64216576	6.07	5.99	5.26		
64216992	6.91	6.83	6.07		
64217122	6.91	6.83	6.06		
64224755	**8.81**	**8.81**	8.07		
64266119	7.26	6.12	5.09		
64266282	7.26	6.12	5.09		
64267101	7.39	6.25	5.09		
Chr05 (*qPC5-2*)					
64580048	**8.50**	**8.53**	7.43	Sobic.005G167600 similar to Pi-b protein Chr05:64628186.64630007 reverse Between 64621753 and 64638422	Not highly expressed
64580306	6.72	6.60	5.45		
64581328	6.81	6.68	5.34		
64581344	6.81	6.68	5.34		
64583869	6.85	6.74	5.56		
64584322	8.06	7.96	7.00		
64584997	6.75	6.66	5.33		
64585014	6.73	6.64	5.33		
64586194	6.63	6.20	6.42		
64586498	7.38	6.84	5.93		
64587305	6.54	6.41	5.89		
64589960	6.96	6.87	5.66		
64590140	6.76	6.66	5.42		
64591940	6.68	6.56	5.44		
64610693	6.77	6.66	5.51		
64612690	6.93	6.86	5.51		
64612943	6.10	6.06	5.05		
64614467	5.44	5.29	5.37		
64616198	6.98	6.84	5.66		
64621753	**11.50**	**11.65**	**9.26**		
64638422	6.41	6.01	5.00		
Chr06 (*qPC6*)					
51113845	3.60	4.51	3.21	Sobic.006G149700 Senescence regulator Chr06:51115119.51116554 reverse Between 51113980 and 51116621	Highest expression in leaf sheath
51113980	**10.48**	**10.36**	**11.15**		
51114635	3.02	3.29	4.43		
51115418	1.79	2.08	4.45		
51115424	2.01	2.32	4.19		
51116621	3.28	4.26	3.09		
Chr09 (*qPC9-1*)					
2824326	**8.97**	**8.96**	**6.59**	Sobic.009G031700 unknown Chr09:2823951.2827282 reverse All three SNPs in coding region	Highly expressed in flowers and leaves
2824605	7.32	7.34	5.16		
2824643	6.43	6.36	5.51		
Chr09 (*qPC9-2*)					
40485401	**8.66**	**8.28**	7.10	Sobic.009G101700 RP-S7e Chr09:40497726.40501826 reverse 16.4 kb from 40485401	Ubiquitously expressed
40485626	7.59	7.20	6.21		
40486124	**8.28**	8.03	6.16		
40487728	6.42	5.99	6.50		
40488163	7.39	7.00	5.83		
40488216	7.92	7.43	5.96		
40491677	6.36	5.91	6.39		
40491733	7.23	6.82	5.82		
40492084	6.29	6.20	4.60		
40492724	7.25	6.86	5.94		
40492742	7.25	6.86	5.94		

*SNP position is based on Sorghum bicolor v3.1.1. −log(P) in bold indicates significance at α = 0.05 or 0.01. Underlined candidate genes are closest to the linked SNPs. ** See **Supplementary Table S2** for expression data.

### 3.3 Candidate gene identification

**Figure 3:**
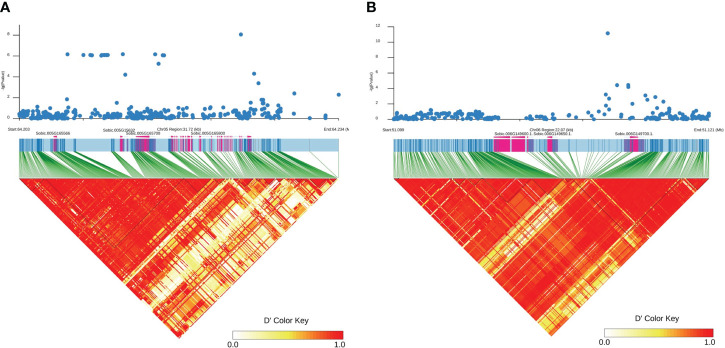
Manhattan plot (top) of the *qPC5-1* **(A)** and *qPC-6* **(B)** loci on chromosomes 5 and 6 and their respective LD blocks (bottom). The X-axis represents position in bp along the chromosome, and the Y-axis is −log_10_(*P*).

Only genes closest to the respectively linked SNPs are presented in **Table 1**. All loci contained one to three candidate genes (**Table 1**). The *qPC5-1* and *qPC6* were further examined with linkage disequilibrium (LD) analysis combined with the Manhattan plot (**Figure 3**). In *qPC5-1*, Sobic.005G165632 and Sobic.005G165700 were located in the same LD block with the QTL peak. In *qPC6*, Sobic.006G149650 and Sobic.006G149700 were located in the different LD blocks. The *qPC6* peak contained only one gene, Sobic.006G149700, which indicates that it should be the candidate gene for *qPC6*. The annotation information showed that Sobic.006G149700 is senescence regulator/heavy metal-associated isoprenylated plant protein 34.

### 3.4 Allelic effect on plant color

**Figure 4:**
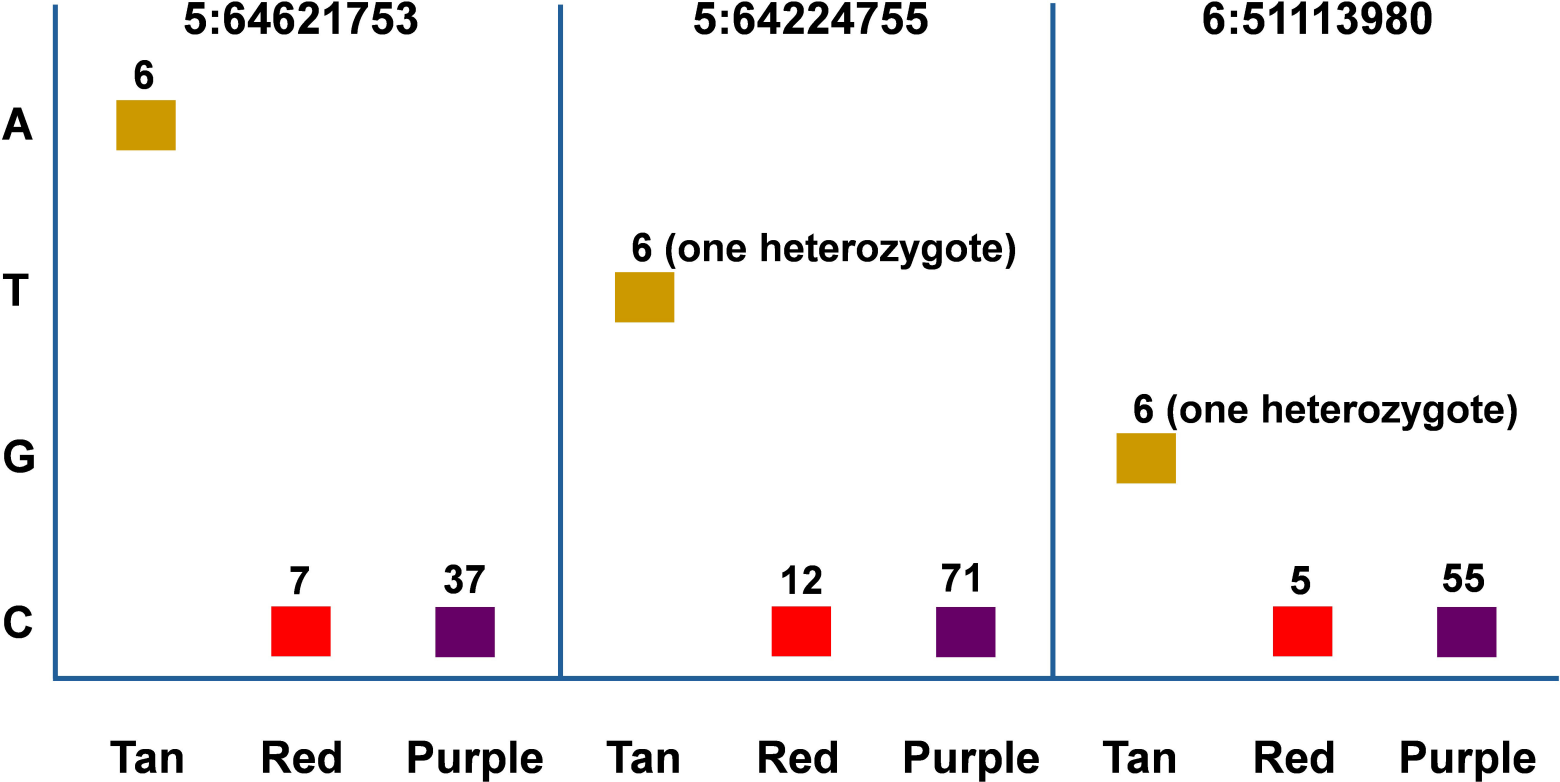
Allelic effect of SNP markers 5:64621753, 5:64224755, and 6:51113980 on sorghum plant color. The accessions with each plant color were selected to maximize the number of each color with minimum missing genotype data rate. Therefore, the accessions with the same color across different SNPs may overlap but may not be identical.

We examined the allelic effect of all SNPs from the eight loci. For each locus, only accessions with missing data rate less than 5% were selected. In all loci, more purple accessions were observed than tan and red combined and no SNPs from the loci could differentiate between purple and red color accessions whereas most SNPs from all loci can differentiate tan from red/purple accessions (**Supplementary Tables S3**-**S8**). We presented three of four SNPs (5:64621753, 5:64224755, and 6:51113980) most tightly linked to plant color from **Table 1** in **Figure 4**. Six tan accessions were identified for all three SNPs whereas 7, 12, and 5 red accessions were identified, respectively. In contrast, 37, 71, and 55 purple accessions were identified respectively for the three markers. In both 5:64224755 (T/C) and 6:51113980 (G/C), IS20740 was the single heterozygote and the T and G alleles respectively were dominant to the C alleles as CC homozygotes in both SNPs were red or purple, whereas the heterozygotes were tan. In the other five accessions, TT and GG genotypes in the two SNPs showed tan plant color. It is coincidental that in all three SNPs, red/purple accessions were all CC genotypes.

## 4 Discussion

White sorghum grain grown on tan plants is highly desirable as livestock feed and for human consumption (Awika et al., 2002). The tan/purple/red plant color is mainly controlled by the *P* and *Q* genes (Dykes et al., 2009; Valencia and Rooney, 2009; Dykes et al., 2011). In this study, we identified eight loci for plant color across three environments. Among these, *qPC6* locus at 51,113,980 bp on chromosome 6 is long way off the plant color QTLs mapped by Boyles et al. (2017). They mapped one locus each at 56650607 and 56635333 bp on chromosome 6 in BTx642/BTxARG-1 and BTxARG-1/P850029 RIL populations, respectively. However, their two QTLs range from 49.9 Mb to 60.77 Mb and from 50.91 Mb to 60.6 Mb, both overlapping with *qPC6*. The peaks at 56,650,607 and 56,635,333 bp are close to the *P* gene (57164448.57187434 in *Sorghum bicolor* v3.1.1), which turns the leaves to purple upon wounding or pathogen invasion (Kawahigashi et al., 2016). This is because Boyles et al. (2017) used *Sorghum bicolor* v3.1 and Kawahigashi et al. (2016) used *Sorghum bicolor* v1.4 at www.plantgdb.org/SbGDB, which is no longer functional at the time of this writing. Therefore, genomic locations are not comparable although Sb06g029550 (Sobic.006G226800) from Kawahigashi et al. (2016) is located in *Sorghum bicolor* v3.1 as from 57,175,961 bp to 57,178,219 bp on chromosome 6. In *qPC6*, those highly associated SNPs were only located in the Sobic.006G149700 gene region (**Figure 3**), which is annotated as a senescence regulator. Its highest expression was in the leaf sheath at floral initiation, followed by seeds at maturity and juvenile leaf blades (**Supplementary Table S2**; McCormick et al., 2018). It is clear that *qPC6* does not overlap with the *P* gene. This could suggest that there are multiple genes responsible for plant color in sorghum. Sobic.006G149700 is orthologous to Arabidopsis *AtS40* (AT2G28400) and its mutation delayed leaf senescence (Fischer-Kilbienski et al., 2010).

As mentioned above, RNA-seq expression data (**Supplementary Table S2**) by McCormick et al. (2018) may help identify candidate genes. In this study, plant color was scored for leaf/leaf sheath color as “1” (tan), “2” (red), or “3” (purple) according to Rooney (2016). Candidate genes physically close to the linked SNPs are either highly expressed in leaves, leaf sheath, or both (**Table 1**; **Supplementary Table S2**). For example, in *qPC1* Sobic.001G324900 is the only gene within 3 kb of the locus and the gene’s highest expression is in the leaves and moderate expression in the leaf sheath; Sobic.002G416400 in *qPC2* is the only gene between the linked SNPs and is highly expressed in the leaf sheath; in *qPC5-1*, three genes are within the locus but only Sobic.005G165800 is highly expressed in both leaves and leaf sheath; as the only gene within the *qPC6* locus, Sobic.006G149700’s highest expression is in the leaf sheath and leaves; and Sobic.009G031700 is the only gene in *qPC9-1* with all linked SNPs in its coding region and is highly expressed in the leaves. The only exception is Sobic.005G167600 in *qPC5-2*, which is the only gene within the linked SNPs, and it is not highly expressed. In contrast, Sobic.004G200700 is 9 kb from *qPC4* and is only highly expressed in the panicles and Sobic.009G101700 in *qPC9-2* is 16 kb away and ubiquitously expressed. These indicate a loose correlation between the degree of linkage and tissue/organ expression of the underlying genes. It is possible that altered expression of these genes could impact plant color scored using leaves and leaf sheath.

Sorghums with red/purple plant color are also induced by fungal attack (Snyder and Nicholson, 1990). In the current study, we also identified one candidate gene associated with fungal resistance. In *qPC5-2*, Sobic.005G165700 is the antimicrobial peptide MBP-1 family protein precursor, which has been reported as effective against both Gram-negative and Gram-positive bacteria as well as several filamentous fungi (Duvick et al., 1992). As stated above, Sobic.005G165800 is highly expressed in both leaves and leaf sheath, although its highest expression is in seed grain at maturity and the panicles (**Supplementary Table S2**). There is no ortholog of this gene in Arabidopsis, and no orthologs in maize or rice have been studied. Therefore, the correlation of plant color and antimicrobial peptide needs to be further investigated.

In conclusion, in this study, we mapped eight loci associated with sorghum plant color, one each on chromosomes 1, 2, 4, and 6 and two on chromosomes 5 and 9. We identified several candidate genes that are highly expressed in the leaves/leaf sheath, and one of the candidate genes was Sobic.006G149700 encoding a senescence regulator. This may facilitate the elucidation of plant color development as well as molecular plant breeding.

## Data availability statement

The original contributions presented in the study are included in the article/**Supplementary Material**. Further inquiries can be directed to the corresponding authors.

## Author contributions

LW: Writing – review & editing. WT: Investigation, Writing – review & editing. PJ: Writing – review & editing. YL: Writing – review & editing. JD: Writing – review & editing. JZ: Writing – review & editing. Y-HW: Writing – original draft. JL: Writing – original draft, Writing – review & editing.
