# Supplementary figures and images for: Genome-wide association study of plant color in Sorghum bicolor
Source: Front Plant Sci. 2024 Apr 10;15:1320844. doi: 10.3389/fpls.2024.1320844 (PMC11039782; doi:10.3389/fpls.2024.1320844)

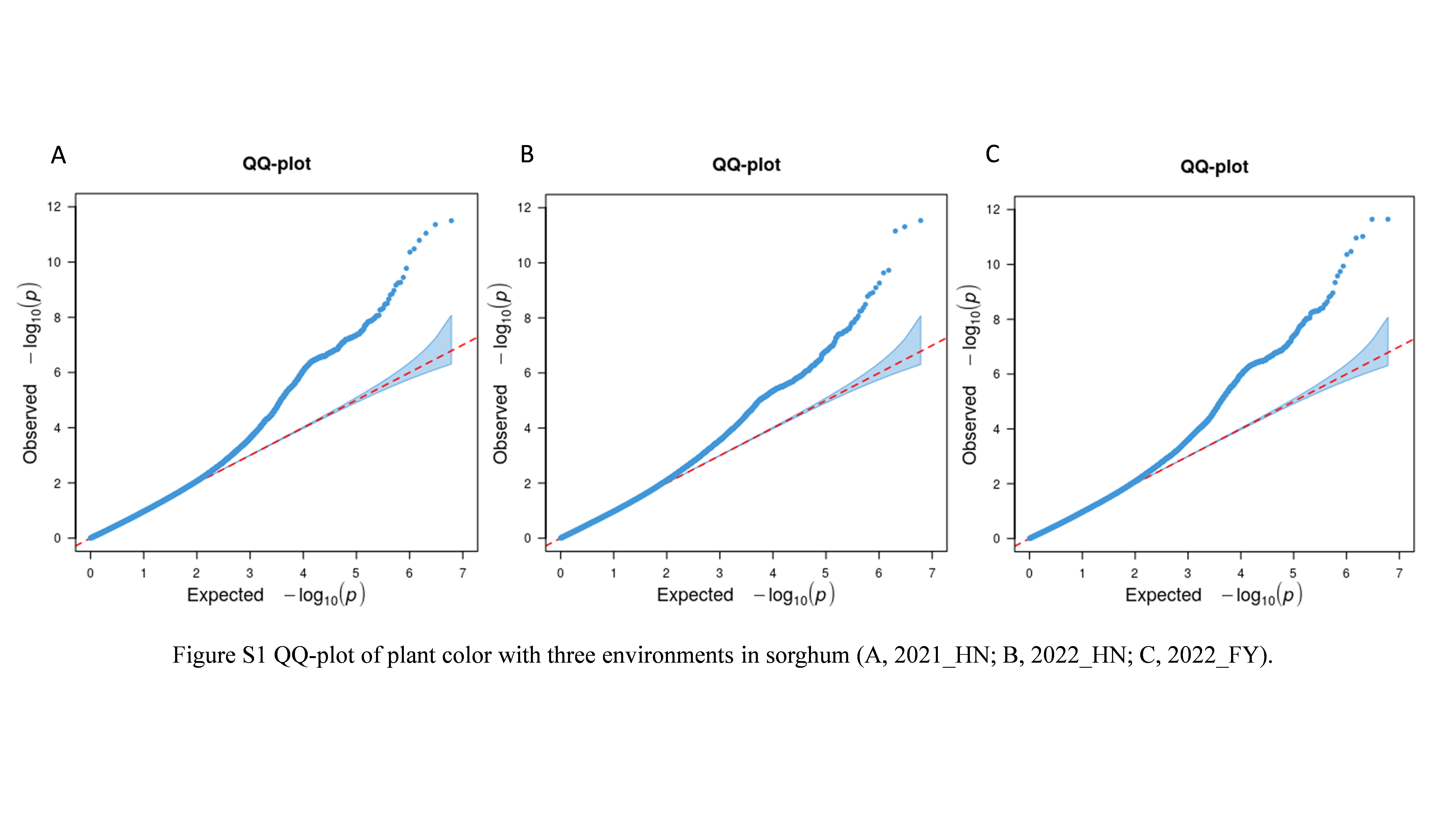

Supplement: Supplementary file 2 [file Image_1.jpeg]
